# Glibenclamide for Brain Contusions: Contextualizing a Promising Clinical Trial Design that Leverages an Imaging-Based TBI Endotype

**DOI:** 10.1007/s13311-023-01389-x

**Published:** 2023-06-12

**Authors:** Ruchira M. Jha, J. Marc Simard

**Affiliations:** 1 Department of Neurology, Barrow Neurological Institute and St. Joseph’s Hospital and Medical Center, Phoenix, AZ USA; 2 Department of Translational Neuroscience, Barrow Neurological Institute and St. Joseph’s Hospital and Medical Center, Phoenix, USA; 3 Department of Neurosurgery, Barrow Neurological Institute and St. Joseph’s Hospital and Medical Center, AZ Phoenix, USA; 4 Department of Neurosurgery, School of Medicine, University of Maryland, Baltimore, MD USA; 5 Department of Pathology, School of Medicine, University of Maryland, Baltimore, MD USA; 6 Department of Physiology, School of Medicine, University of Maryland, Baltimore, MD USA

## Abstract

TBI heterogeneity is recognized as a major impediment to successful translation of therapies that could improve morbidity and mortality after injury. This heterogeneity exists on multiple levels including primary injury, secondary injury/host-response, and recovery. One widely accepted type of primary-injury related heterogeneity is pathoanatomic—the intracranial compartment that is predominantly affected, which can include any combination of subdural, subarachnoid, intraparenchymal, diffuse axonal, intraventricular and epidural hemorrhages. Intraparenchymal contusions carry the highest risk for progression. Contusion expansion is one of the most important drivers of death and disability after TBI. Over the past decade, there has been increasing evidence of the role of the sulfonylurea-receptor 1–transient receptor potential melastatin 4 (SUR1-TRPM4) channel in secondary injury after TBI, including progression of both cerebral edema and intraparenchymal hemorrhage. Inhibition of SUR1-TRPM4 with glibenclamide has shown promising results in preclinical models of contusional TBI with benefits against cerebral edema, secondary hemorrhage progression of the contusion, and improved functional outcome. Early-stage human research supports the key role of this pathway in contusion expansion and suggests a benefit with glibenclamide inhibition. ASTRAL is an ongoing international multi-center double blind multidose placebo-controlled phase-II clinical trial evaluating the safety and efficacy of an intravenous formulation of glibenclamide (BIIB093). ASTRAL is a unique and innovative study that addresses TBI heterogeneity by limiting enrollment to patients with the TBI pathoanatomic endotype of brain contusion and using contusion-expansion (a mechanistically linked secondary injury) as its primary outcome. Both criteria are consistent with the strong supporting preclinical and molecular data. In this narrative review, we contextualize the development and design of ASTRAL, including the need to address TBI heterogeneity, the scientific rationale underlying the focus on brain contusions and contusion-expansion, and the preclinical and clinical data supporting benefit of SUR1-TRPM4 inhibition in this specific endotype. Within this framework, we summarize the current study design of ASTRAL which is sponsored by Biogen and actively enrolling with a goal of 160 participants.

## Introduction

Traumatic brain injury (TBI) is projected to remain the leading cause of neurological disability in 2030. Its current global incidence of 50 million outpaces diseases such as stroke and Alzheimer’s [1]. Over the past several decades, promising high-profile therapies in TBI have failed to translate to human benefit including steroids, progesterone, hypothermia, magnesium, and erythropoetin [2–8]. In large part, this has been attributed to challenges of disease heterogeneity versus treatment homogeneity [9, 10]. In this article we present the background, scientific rationale and study design for a unique ongoing multicenter phase-II clinical trial that attempts to tackle the problem of TBI heterogeneity while evaluating an intravenous formulation of glibenclamide: ASTRAL ($\underline{A}$ntagonizing $\underline{S}$UR1-$\underline{TR}$PM4 to reduce the progression of intr$\underline{A}$cerebral hematoma and edema surrounding $\underline{L}$esions). This trial is a first of its kind in TBI in that it leverages robust underlying pathobiology to address TBI heterogeneity by focusing both its patient population and primary outcome to a pathoanatomic endotype of TBI: brain contusion. Brain contusions are an imaging-based TBI endotype that are comprised of an intraparenchymal hematoma and surrounding/perilesional edema. High-specificity patient selection of this TBI subpopulation identifies those most likely to benefit from glibenclamide based on strongly supportive preclinical data. The primary outcome in ASTRAL is contusion expansion which includes progression of the intraparenchymal hematoma component as well as the perihematomal edema. This imaging-based outcome is mechanistically linked to the molecular process targeted by glibenclamide. Importantly, it is also a key death and disability-driving secondary injury in patients with contusional-TBI.

## TBI Heterogeneity and the Need for Endotyping

TBI heterogeneity develops on impact and persists through recovery. In reality, this is also compounded by pre-injury heterogeneity in baseline brain health/cerebral constitution including factors like cognitive reserve, resilience, frailty, pre-morbid psychiatric disorders— all of which influence outcome after TBI and have been increasingly appreciated in recent research [11–14]. The initial impact in TBI instigates a cascade of unique secondary injury responses that depend on a variety of factors including both host- (age, sex, co-morbidities) and injury-characteristics (mechanism, severity velocity, force, intracranial compartment). Implications of pathoanatomic differences (epidural, subdural, subarachnoid, intraparenchymal, diffuse axonal injuries) may be distinct in terms of downstream complications that are clinically discernable (e.g., seizures, hemorrhage progression/contusion expansion, intracranial hypertension, need for neurosurgical intervention) as well as a molecularly important (e.g., neuroinflammation, energy failure, neurodegeneration, cell-death). It follows that therapeutic needs of these subpopulations may also be unique. Indeed, the existing TBI treatment “one-size-fits-all” standardized/protocolized [15] approach is increasingly being recognized as suboptimal [16–18]. Although several clinical trials have attempted to account for TBI heterogeneity using a severity-based stratification [19, 20], this approach does not account for key pathoanatomical and molecular differences between different types of primary injury and thus does not facilitate targeting specific pathobiology. This has been a key criticism of the traditional Glasgow Coma Scale symptom-based classification [21, 22].

The concept of endotyping, initially defined in asthma with T2-helper eosinophilic/interleukin-5 signaling responsive vs non-T2 subtypes, is now leveraged in several fields (oncology, sepsis, pulmonology) and is highly relevant to TBI. It is the principle that specific biological mechanisms/pathways culminate in precise clinical phenotypes such as contusion expansion [23, 24]. Therapies may have limited success if generically applied to populations where the pathway is only active in a subgroup [23, 24]. TBI molecular endotyping is in its infancy but is growing and essential to advance therapy for secondary injury. Successful TBI endotyping could guide clinicians to identify both the correct target mechanism (to monitor or treat), and the correct patient population. This is valuable for trials and ultimately bedside TBI care. Imaging characteristics and biofluid markers have gained traction in research efforts to improve TBI endotyping [22]. They have distinct but complementary utilities. The blood biomarkers closest to implementation in clinical practice are the combination of glial fibrillary acidic protein and ubiquitin C-terminal hydrolase L1, which are primarily diagnostic (in terms of predicting computed tomography [CT] and magnetic resonance imaging [MRI] positivity) [25–27]. These particular biomarkers, however, have been more useful in determining overall burden of injury and prognostication (particularly in severe TBI) rather than being able to differentiate pathoanatomical types of TBI [22, 28–32]. Imaging thus remains key for spatial and compartmental endotyping in TBI particularly given the potential treatment and prognostic implications.

## Contusional TBI—A Targetable Major Endotype of Morbidity and Mortality

Traumatic intraparenchymal cerebral contusions are a subgroup of TBI that can be clearly and practically defined on non-contrast head CTs. They occur in ~ 35% of moderate-severe TBI cases and are associated with unfavorable outcome and mortality [33–36]. Contusion expansion, constituting progression of both cerebral edema and hemorrhage, occurs in approximately 50% of patients (16–75%) [33–35, 37, 38]. The hemorrhage progression component most frequently occurs within the first 12–24 h [33, 34, 39]. Although not universal, this clinical decompensation (often requiring neurosurgical intervention) has been identified in a large number of studies as a key contributor to secondary injury, disability, and death; the odds of unfavorable outcome reported have been as high as 5X, and mortality can be more than threefold increased [34, 39–49].

The wide range of secondary hemorrhage progression incidence in contusional TBI reported in the literature may be partly attributable to variability in definition [33]. These definitions for progression and ensuing risk-factor identification are relevant to understanding the study design and patient selection for ASTRAL. Most studies evaluating the impact of traumatic secondary hemorrhage progression of a contusion on various outcomes defined progression as a ~ 30–33% increase from initial volume [34, 46–48, 50–57]. However, others included “any” increase [37, 45, 49, 58–60], an increase of 5% [61], or absolute volume changes (of 1 cm^3^ or 12.5 mL) [43, 62].

### Risk and Effects of Contusional Hemorrhage Progression

Risk factors for hemorrhage progression of a contusion in the aforementioned studies have been reported with varying robustness. They include an extensive range of clinical, laboratory, and imaging characteristics such as injury severity (as measured by the Glasgow Coma Scale (GCS) score), history of hypertensive disease, initial contusion volume, contusion location, presence of peri-hematomal edema, co-existing lesions (especially subdural and subarachnoid hemorrhages), skull fractures, contrast extravasation, hypoxia, decompressive craniectomy, coagulopathy, and low triglyceride levels [33]. Certain patient populations are particularly vulnerable to contusional hemorrhage progression like the elderly, those on therapeutic anticoagulation, and chronic alcoholics [63–67]. Collinearity between some of these variables (such as advanced age and therapeutic anticoagulation) makes it challenging to dissect independent influence on hemorrhage progression, but there are likely biologically distinct effects on vessel fragility versus impaired hemostatic ability.

For some “risk” factors of contusional hemorrhage progression (such as post-traumatic coagulopathies), the data have been controversial. However, others have been largely consistent in multiple independent evaluations over more than a decade. Initial contusion volume in particular has been a reproducibly robust risk factor associated with contusion expansion [37, 45, 50, 53, 54, 57, 58, 68] with one exception [47]. One study of 113 TBI patients quantified this risk where each additional cm^3^ of contusion volume increased the risk of expansion by 11%. Another study in 246 patients identified an increased odds ratio of ~ 5 for contusion volumes > 20 mL [50]. A majority of the prior studies confirms the negative impact of secondary hemorrhage progression on several outcomes (neurosurgical intervention, mortality, functional outcome) [38–41, 43, 45, 49, 53–55, 57–59, 69–71]. Even studies where hemorrhagic progression of a contusion was defined as “any” increase (vs the more commonly used definition of > 30–33%) reported a largely consistent association with unfavorable outcome, including a large prospective analysis of 1200 patients [45, 49, 58–60]. This is not surprising given the long-recognized toxicity of extravasated blood to brain cells and impact of associated hemorrhagic necrosis beyond just mass effect [72].

### Mechanisms Underlying Contusional Hemorrhage Progression

Since most secondary hemorrhage progression in contusional-TBI occurs within 12–24 h, there is a window of opportunity for prevention. However, to date no therapies have demonstrated success at inhibiting this process and improving outcomes in human patients. Conventionally, coagulopathies have been considered important mechanistic contributors to progressive hemorrhage of already injured microvessels. However, this concept remains unproven, particularly given the mixed data. Coagulopathies are defined broadly in TBI and can include perturbations in any single coagulation parameter, including prolonged prothrombin time (PT), activated thromboplastin time (aPTT), elevated international normalized ratio (INT), or decreased platelet counts. Although common in TBI (15–100% depending on injury severity), coagulation abnormalities are not necessary in the causal pathway of secondary hemorrhage progression. Of patients with secondary hemorrhage progression of their contusion, only 3–31% have abnormal coagulation parameters (PT, PTT), and of patients with abnormal coagulation parameters, 40–100% have secondary hemorrhage progression [39, 41, 44, 71]. Despite the 1% reduction in mortality (limited to mild-moderate TBI), it is therefore not surprising that tranexamic acid had no impact on hemorrhage progression in two large randomized controlled trials [73, 74].

**Fig. 1:**
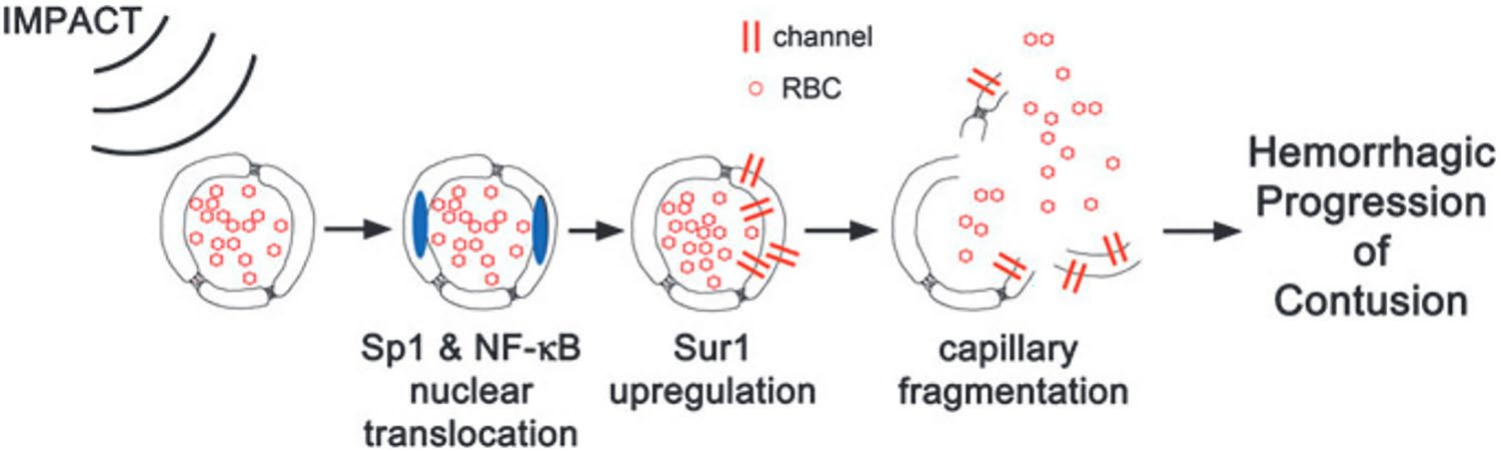
Schematic demonstration of molecular changes that are proposed to contribute to hemorrhagic progression of a contusion. The kinetic energy from primary impact activates key mechanosensitive transcription factors like specificity protein-1 and nuclear factor-kb. These directly result in transcriptional upregulation of the Sur1-Trpm4 channel in endothelial cells that result in cellular edema and oncotic cell death- which ultimately contributes to breakdown of capillary integrity and hemorrhagic progression of the original contusion. (Adapted with permission from Kurland et al. [38])

An alternative hypothesis for the mechanism underlying secondary hemorrhage progression of a contusion has been developed over the past decade and involves the concept of progressive microvascular failure in penumbral tissue [38, 75, 76]. This theory is supported by early data and provides an explanation for how microvessels that are not affected during primary injury end up facilitating extravasation of blood several hours later [38, 75, 76]. These studies demonstrate that the initial kinetic energy from focal impact may not shear tissue/microvessels in the contusional penumbra but are sufficient to activate mechanosensitive molecular processes (specificity protein1 [Sp1], and nuclear factor-kB [NF-kB]). These in turn initiate a series of events in the penumbra that result in delayed structural failure of the microvasculature including smooth muscle and endothelial cells, which is responsible for hemorrhagic progression of the contusion (Fig. 1). A key downstream channel upregulated by these mechanosensitive processes that has been demonstrated in recent research to be central to secondary hemorrhage progression of a contusion, is sulfonylurea-receptor 1–transient receptor potential melastatin 4 (SUR1-TRPM4) [34, 35, 75–84]. Channel inhibition has yielded promising results, particularly for brain contusion as discussed below.

## Research Supporting Benefit of Glibenclamide in Contusional-TBI

The SUR1-TRPM4 channel was first identified in 2001 as a non-selective cation channel regulated by internal calcium and adenosine triphosphate (ATP) in reactive astrocytes cultured from rat brain, where activation led to sodium influx, cellular swelling, and blebbing [85]. Five years later, its role in cerebral edema development after massive middle cerebral artery infarction (ischemic stroke) and the benefit of channel inhibition with glibenclamide were demonstrated in rats [86]. Glibenclamide is a second generation sulfonylurea that inhibits SUR1-TRPM4 by increasing the probability of long-closed state of the channel without impacting open channel dwell times or conductance [78]. Although studies of SUR1-TRPM4 and glibenclamide in ischemic stroke initially dominated the literature, there has been increasing evidence of its centrality to secondary injury after TBI, particularly as it relates to contusional edema and hemorrhage progression (Table 1) [35, 78].

**Table 1:** Studies of Glibenclamide in Contusional TBI

**A: Preclinical studies in controlled cortical impact**			
*Authors, year*	*Model*	*Glibenclamide dose*	*Glibenclamide impact on outcome*
Simard et al. (2009)	Rat focal cortical contusion (4.5 mm impactor, 10 g weight, 5 cm drop, 1 m/s velocity)	Loading: 10 μg/kg IP Maintenance: 200 ng/h Timing: 10 min of TBI	↓ hemorrhage expansion (minimal) at 6 h, 12 h, 24 h ↓ 24 h histological lesion volume Improved spontaneous vertical exploration
Patel et al. (2010)	Rat CCI (Modified Feeney, 10 g weight, 3 cm drop, 0.7 m/s)	Loading: 10 μg /kg IP Maintenance: 200 ng/h (7 days) Timing: 10 min of TBI	↓ hippocampal caspase-3 Improved hidden platform trial (memory retention) 3X ↓ in FJC + hilar cell death
Zweckberger et al. (2014)	Rat CCI (5 mm impactor,1.5 mm depth, 7.5 m/s, 300 ms duration)	Loading: 10 μg/kg IP Maintenance: 200 ng/h Timing: 15 min after TBI	No improvement in intracranial pressure, cerebral microdialysis parameters, seizures, or beam walk ↓ 24 h brain water content (ipsilateral) by 15.3% ↓ contusion volume at 8, 24, 72 h, and 7 days
Xu et al. (2016)	Mouse CCI (3 mm impactor,1.5 mm depth,1.5 m/s duration)	10 μg /day (3 days) Timing: immediately after TBI	↓ 72 h ipsilateral brain water by 51.6% ↓ BBB disruption ↓ loss of ZO-1 ↓ early stage apoptosis induced by stretch injury
Jha et al. (2018)	Mouse CCI (1 mm depth, 5 m/s) ± HS (MAP 25–27 mmHg, 20 min)	Loading: 20 μg /kg IP Maintenance: 400 ng/h Timing: 10 min after TBI	↓ contralateral brain water to sham levels after CCI + HS
Gerzanich et al. (2019)	Rat CCI (5 mm impactor, 4.5 mm depth, 1 m/s, 200 ms duration)	Loading: 10 μg /kg IP Maintenance: 400 ng/h Timing: 10 min after TBI	↓ hemorrhagic progression of contusion by 58% ↓ mean swelling by ~ 66%
Jha et al. (2021)	Rat CCI (8 mm impactor, 2.6 mm depth, 4.5 m/s)	Loading: 10 μg /kg IP Maintenance: 200 ng/h (7 days) Timing: 10 min after TBI	Improved motor function (beam balance, walk) ↓ 21d contusion volume
Zusman et al. (2023)	Mouse CCI (0.5 mm depth, 4 m/s) ± HS with MAP 25–27 mmHg X 20 min	High Dose: 10 mg/mouse Low Dose: 20 mg/kg Maintenance: 400 ng/h (7 days) Timing: 10 min after TBI or within 5 min of HS	↓ hematoma volume, vasogenic edema, cytotoxic edema, BBB integrity in CCI → high dose only ↓ hematoma volume in CCI + HS → high dose only No benefit with low dose

**B: Clinical studies in contusional TBI**			
*Authors, year*	*Design*	*Dose and sample size*	*Results*
Khalili et al. (2017)	Double-blind randomized controlled trial, moderate-severe TBI (GCS 5–12)	10 mg/day oral GLI (10 days) Timing: first dose within 10 h of TBI N = 66	No difference in contusion volume between GLI vs placebo at baseline, day 3, and day 7 ↓ contusion volume expansion ratio between day 1 vs 3 (p < 0.001) and day 1 vs 7 (*p* = 0.003) No difference in functional outcome at 3 months as measured by GOS, MRS, or DRS
Eisenberg et al. (2019)	Phase-2 Randomized Controlled Trial	BIIB093 (intravenous GLI) IV 0.13 mg load over 2 min, 0.16 mg/h for 6 h, 0.11 mg/hr for 66 h Timing: within 10 h of TBI *N* = 28 (*N* = 14 contusion)	↑ Contusion growth (non-significantly, tenfold) in placebo (1036 ± 1963.28%) vs GLI (136 ± 195.62%; *p* = 0.15, *n* = 7/group) MRI measures of edema (FW, MD, MDt) in lesion vs uninjured WM were different in placebo (p < 0.02) but not GLI

*BBB* blood brain barrier, *CCI* controlled cortical impact, *FJC* Fluoro Jade-C, *FPI* fluid percussion injury, *GLI* glibenclamide, *PBBI* penetrating ballistic-like brain injury, *SUR1* sulfonylurea receptor 1, *TBI* traumatic brain injury, *ZO-1* zona occludens-1, ↓ decrease

The first report identifying the key role of SUR1 in secondary hemorrhage progression of a contusion was in a rat model of focal cortical contusion [75]. This was pivotal in defining early (within 3 h) SUR1 upregulation in microvessels that persisted for 24h [75]. Glibenclamide reduction of hemorrhage progression emerged within 45 min, and was sustained at 3 h, 6 h with maximal benefit at 12 h. Several subsequent studies in preclinical contusional TBI across different models, severities, and species have consistently shown that glibenclamide improves several outcome measures including contusion expansion, cerebral edema, blood-brain-barrier integrity, and functional outcomes, both motor and cognitive [75–77, 80, 81, 83, 84, 87]. This includes testing by the rigorous, blinded, multi-center preclinical consortium, Operation Brain Trauma Therapy (OBTT) where most drugs perform significantly “below expectations based on the published literature.” [88] OBTT tested glibenclamide in three models of TBI—controlled cortical impact (CCI, a model of contusional TBI), fluid percussion injury, and blast injury—with specific benefit detected in CCI [81]. Indeed, glibenclamide is the only drug to have ever been tested by OBTT to reduce contusion volume in CCI, and it is the second highest scoring drug overall (after levetiracetam). Both the anti-edema benefits of glibenclamide after contusional TBI and the reduction of hemorrhage progression have also been confirmed by multiple independent laboratories using different models and outcome measures [35, 75–78, 80, 83, 84, 87].

Early treatment is likely essential, particularly for limiting the hemorrhage progression component of contusion expansion since upregulation of SUR1-TRPM4 in the endothelium occurs as early as 3h [75, 77]. A recent murine study in CCI evaluating the impact of glibenclamide on MRI-based endotypes confirmed that the reduction in hematoma volume (and diffusion-restrictive cellular edema) occurred acutely with high-dose treatment; however, the impact on vasogenic edema had a longer time-window [80]. Combining these results with the known delayed/persistent SUR1-TRPM4 expression in astrocytes and microglia, continued or even later-initiated treatment with glibenclamide may decrease some edema formation and/or neuroinflammatory processes. Aside from treatment time, dosing (and injury pattern) may be key. In murine studies, although a lower loading dose of 20 mg/kg achieved steady-state pharmacokinetic serum levels of ~ 10 ng/mL [87], only the higher-dose of 10 mg/mouse effectively reduced hematoma volume, cytotoxic edema, vasogenic edema, and improved blood-brain barrier integrity measured on MRI [80]. It is unclear how these murine doses will translate to those used in the ongoing phase-II clinical trial discussed below. Additional patient/injury characteristics may also impact benefit. At least in the murine study, the addition of hemorrhagic shock appeared to attenuate the anti-edema benefit, but the reduction of secondary hemorrhage progression of the contusion remained robust.

Human studies support the role of SUR1-TRPM4 and glibenclamide in TBI, particularly contusional TBI [34, 77, 79, 82, 89–95] (Table 1B). SUR1 ± TRPM4 expression (and colocalization) has been detected in human brain contusions. Although expression levels have varied by cell type and distance from the epicenter/contusional core, these studies confirm early microvascular expression that has been reported to persist up to 100h [77, 79]. A prospective observational targeted gene study in 321 patients identified eight genetic variants in *ABCC8* (SUR1 gene) and *TRPM4* that were expression quantitative trait loci (eQTL) [34]. These polymorphisms were associated with risk of hemorrhage progression in a directionally and biologically consistent manner (i.e. eQTLs that increased mRNA had an increased risk of hemorrhage progression) [34]. Oral glibenclamide has also been tested in a small randomized placebo controlled trial of 66 patients with traumatic brain contusion [82]. Although oral glibenclamide does not lend itself to tight control of maintaining therapeutic levels, this study did identify lower contusion expansion ratios between baseline-to days 3 and 7. An intravenous formulation of glibenclamide (currently known as BIIB093, previously known as Cirara or RP 1127) was also tested in a small pilot randomized trial of 28 patients with TBI [89] with a subset of 14 patients with contusion. In this study, lesion volume increased 1036% with placebo vs 136% with glibenclamide; however, this finding was not significant possibly due to the small sample size.

## ASTRAL Trial Design—Leveraging Underlying Biology and the Contusional-TBI Endotype

Since the discovery and report of the SUR1-TRPM4 channel two decades ago, there has clearly been a steady stream of molecular, translational, and early clinical research from independent laboratories that supports the likelihood of glibenclamide benefit, particularly in the contusional endotype of TBI. These compelling data propelled enthusiasm to test this therapy in a larger phase-II randomized clinical trial, ASTRAL (NCT03954041). ASTRAL uses a proprietary intravenous formulation of glibenclamide (BIIB093) [35]. In addition to the promising foundational research, this study leverages knowledge gained from previous clinical TBI trials and addresses two key deficiencies: (1) it accounts for one key component of TBI heterogeneity by focusing on a single pathoanatomical endotype, i.e., brain contusion; and (2) it uses a primary endpoint, i.e., contusion expansion (hematoma plus perihematomal edema) that is mechanistically linked to the action of the drug and the known molecular pathobiology described above.

ASTRAL is sponsored by Biogen and is actively enrolling patients. It is an international multi-center double blind multidose placebo-controlled phase-II clinical trial with sites in the United States, Japan, Spain, France, Germany, Israel, and Italy. The enrollment goal for the study is 160 patients. The inclusion and exclusion criteria (Table 2) aim to enroll adult TBI patients with supratentorial brain contusions that are expected to survive for at least 24 h. Setting a minimum size of enrollable contusion (3 mL) is consistent with previously published findings that the risk of contusion expansion is strongly correlated with initial contusion volume, and one report indicating that contusions ≤ 4 mL are unlikely to progress with a sensitivity of 95% and specificity of 75% [53].

**Table 2:** Current key inclusion and exclusion criteria for ASTRAL

**Inclusion**	**Exclusion**
- Age 18 to 85 (male and female adults). - Supratentorial brain contusion with intraparenchymal lesions totaling > 3 mL per investigator assessment of the baseline non-contrast CT. - Glasgow Coma Scale score 5–15. - Functionally independent prior to TBI.	- Anticipated withdrawal of care within 24 h as determined by the site investigator. - Indication for immediate decompressive craniectomy or evacuation of intraparenchymal hematoma. - Clinical signs of brainstem herniation based on the site investigator’s assessment. - Evidence on CT or MRI of penetrating brain trauma from external object such as bullet. - Any presence of midbrain or posterior fossa injury as assessed by the site investigator. - Life threatening or non-survivable polytrauma as assessed by the site investigator. - Known use of oral anticoagulants including direct thrombin inhibitors, or factor Xa inhibitors in 3 days preceding the injury.

**Fig. 2:**
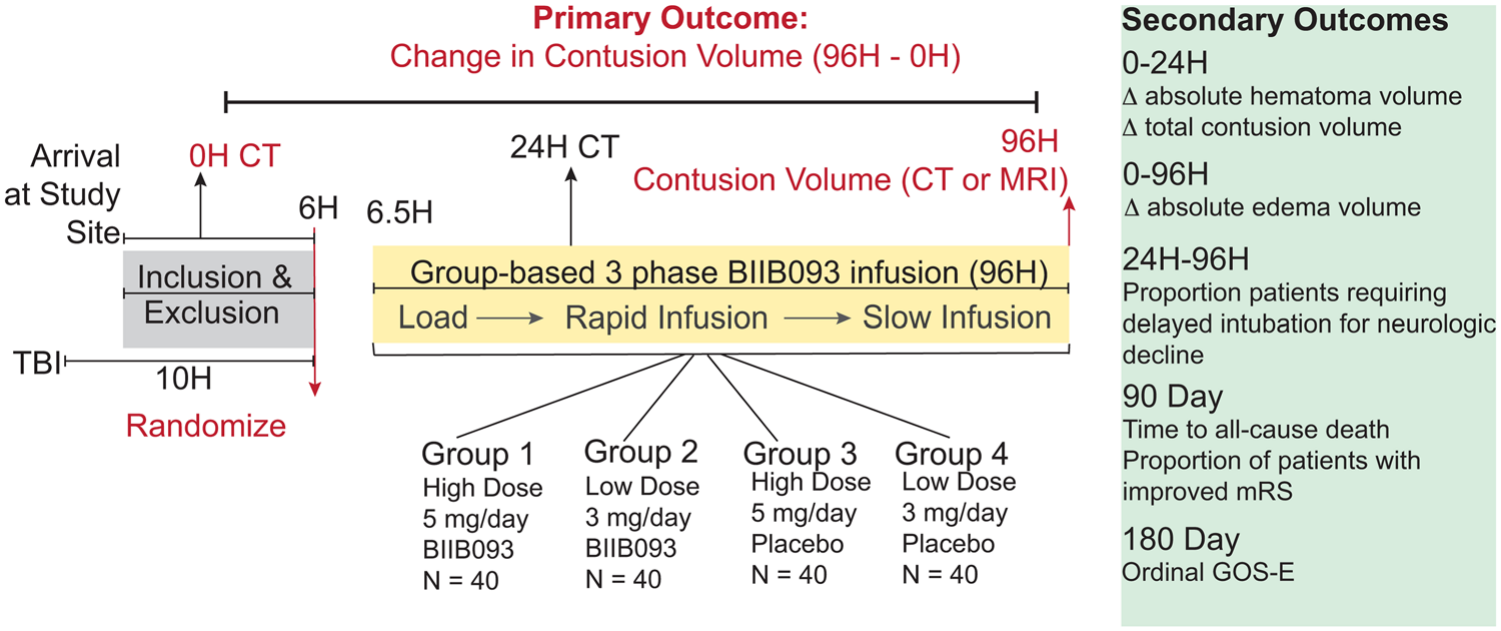
Schematic of the current clinical trial design of ASTRAL: a phase-2 multicenter, international double-blind, placebo-controlled, multi dose randomized clinical trial evaluating the safety and efficacy of BIIB093 in contusional-traumatic brain injury. The timeline from injury to randomization is presented (10 h), and within 6 h of presentation to the study site. Participants are randomized into four groups: low vs high dose BIIB093 and low vs high dose placebo. Drug or placebo need to be infused within 6.5 h of arrival to the study site.The primary endpoint is change in contusion volume from baseline CT (on admission) versus 96-h imaging (either CT or MRI), with several secondary endpoints also being evaluated

The details presented herein about the ASTRAL protocol are accurate as of March 2023 but may evolve as the trial and enrollment progresses. Four study arms have been included (Fig. 2): low dose (3 mg/day) and high dose (5 mg/day) for 96 h, and matched placebo groups. Randomization is required to begin within 6 h of injury, with drug (or placebo) infusion by 6.5 h. Like the inclusion/exclusion criteria, the dose and timing of drug infusion also appear to have been carefully considered in the context of the preexisting research. The safety low dose (~ 3 mg/day) is the same as that used in the phase-1 (and II) study of the same drug in large hemispheric infarction [96, 97]. Pharmacokinetic data from this trial are eagerly awaited – it will be interesting to see how the high and low dose of BIIB093 in this clinical trial compare with the recent murine study demonstrating high-dose-only benefit, since it is challenging to translate drug doses across species. Unlike the infusion timing in large-hemispheric infarction and the prior Eisenberg study in TBI, the directive in ASTRAL is to infuse drug within 6.5 h of injury, given the earlier upregulation of SUR1-TRPM4 in the penumbral microvessels after contusional TBI. The goal is to inhibit microvascular SUR1-TRPM4 before the ensuing oncotic cell death, structural failure, pericontusional edema, and hemorrhage progression.

The primary outcome of ASTRAL is the change in contusion volume from baseline to 96 h, as measured on imaging, either MRI or non-contrast CT. Contusion volumes include the sum of both the hematoma and perihematomal edema. Absolute volumes of the individual components of the contusion will also be recorded and measured as secondary outcomes, including the change in absolute hematoma volume by 24 h from baseline, and the change in absolute edema volume by 96 h from baseline. These distinct timings are related to the peak timing for hemorrhage progression (24 h) versus cerebral edema (3–5 days) that also coincide with the temporal course of SUR1-TRPM4 expression in different cell-types driving either hemorrhage progression (microvascular cells) or cerebral edema (including neurons, glia). Of note, these outcome measures have been updated from our previous report where contusion expansion was defined as a binary measure and the outcome was the proportion of patients with expansion by 96 h. If participants undergo decompressive craniectomy, intraparenchymal hematoma evacuation, or transition toward comfort measures, the scan immediately prior will be used for comparison to baseline. Other important secondary outcome measures that will be obtained in ASTRAL include the proportion of patients requiring delayed intubation (between 24 and 96 h post-injury), the proportion of patients with improved modified Rankin scale by 90 days, as well as overall survival at 90 days and functional outcome measured by an ordinal Glasgow Outcome Scale-Extended score at 180 days. Based on the current rate of enrollment and the sample size needed to power the study, ASTRAL is expected to be completed with final data collection for the primary outcome measure by January 2025.

## Conclusions

Patients with brain contusion (a pathoanatomic endotype of TBI) are at risk for the devastating secondary injury process of contusion expansion. No therapies are currently available to prevent or treat this process. Preclinical and molecular research over the past decade strongly implicates a key role of the SUR1-TRPM4 channel in secondary injury and contusion expansion with benefit of glibenclamide. ASTRAL is an actively enrolling innovative phase-II randomized clinical trial evaluating the safety and efficacy of intravenous glibenclamide in contusional TBI. It addresses the “Achilles heel” of TBI trials, i.e., heterogeneity by focusing on brain contusion (the subpopulation most likely to benefit) and an outcome measure (contusion-expansion) that is both mechanistically linked to glibenclamide and a key driver of morbidity and mortality. It is a first and exciting step toward executing an endotype-driven precision medicine trial in TBI with results anticipated in 2025. Ultimately, an integrative multimodal approach to brain contusion may combine targetable mechanisms with both molecular- and imaging-based endotyping, including biofluid and genetic markers to truly bring precision medicine to the bedside in TBI. Given the strength of the underlying science, developing and validating such tools for the SUR1-TRPM4 pathway may be particularly rewarding.
